# Post nirmatrelvir/ritonavir erythema multiforme in a patient with coronavirus disease infection

**DOI:** 10.1590/0037-8682-0008-2023

**Published:** 2023-06-02

**Authors:** José Wagner Leonel Tavares-Júnior, José Carlos Jucá Pompeu, Luiz Eduardo Garcia Galvão

**Affiliations:** 1 Universidade Federal do Ceará, Fortaleza, CE, Brasil.; 2 Universidade de Fortaleza, Fortaleza, CE, Brasil.; 3 Centro de Dermatologia Dona Libânia, Fortaleza, CE, Brasil.

**Keywords:** COVID-19, Nirmatrelvir/ritonavir, Erythema multiforme

## Abstract

Erythema multiforme (EM), an immune-mediated skin condition, can occur after infection or following the use of medications. In this study, we describe a patient who developed EM after nirmatrelvir/ritonavir administration. An 81-year-old woman presented with fever and dyspnea. Laboratory investigations showed positive coronavirus disease (COVID-19) based on polymerase chain reaction assay, and she received a 5-day regimen of nirmatrelvir/ritonavir. We observed development of EM after this treatment and initiated prednisone (1 mg/kg) therapy, which led to rapid improvement. Our study is the first to report EM in a patient with COVID-19, who received nirmatrelvir/ritonavir and showed a favorable response.

## INTRODUCTION

Erythema multiforme (EM), an immune-mediated condition involving the skin and mucous membranes^1^ ^,^ ^2^, has an estimated prevalence of less than 1%^3^. EM predominantly affects women and adults aged 20-40 years^1^. The pathophysiology of this condition involves the production of immunocomplexes, which can be precipitated by infections or medications and injure small vessels in the skin and mucous membranes^4^. Lesions may be asymptomatic or may present with dysesthesia, and the mean duration varies from 1 to 4 weeks. EM is characterized by target lesions, which may present as macules, papules, or vesicles, mainly distributed on the hands, feet, knees, and elbows^1^ ^,^ ^5^. Treatment involves withdrawal of the possible causative agent in addition to corticosteroid therapy^1^. 

Coronavirus disease (COVID-19), an infection caused by the severe acute respiratory syndrome coronavirus 2^6^ may present with cutaneous manifestations^7^. Antiviral medications, including nirmatrelvir/ritonavir are approved for treatment in patients at risk of adverse clinical evolution^8^. 

In this article, we describe a patient who developed EM after nirmatrelvir/ritonavir administration.

## CASE REPORT

We searched the MEDLINE database via PubMed without any time restrictions. The following descriptors (bold), synonyms, natural language, and Boolean operators were used to cross-check the databases: MEDLINE (Medical Subject Headings [MeSH]: search strategy (nirmatrelvir/ritonavir), (COVID-19), and (erythema multiforme). The search was performed on January 19, 2023.

**FIGURE 1:**
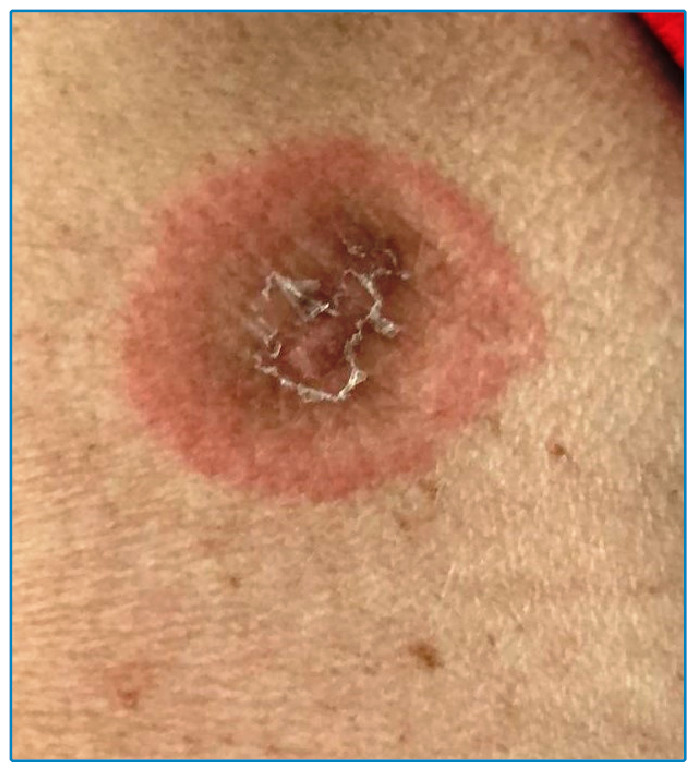
Photograph showing an erythematous maculopapular “target” lesion.

**FIGURE 2:**
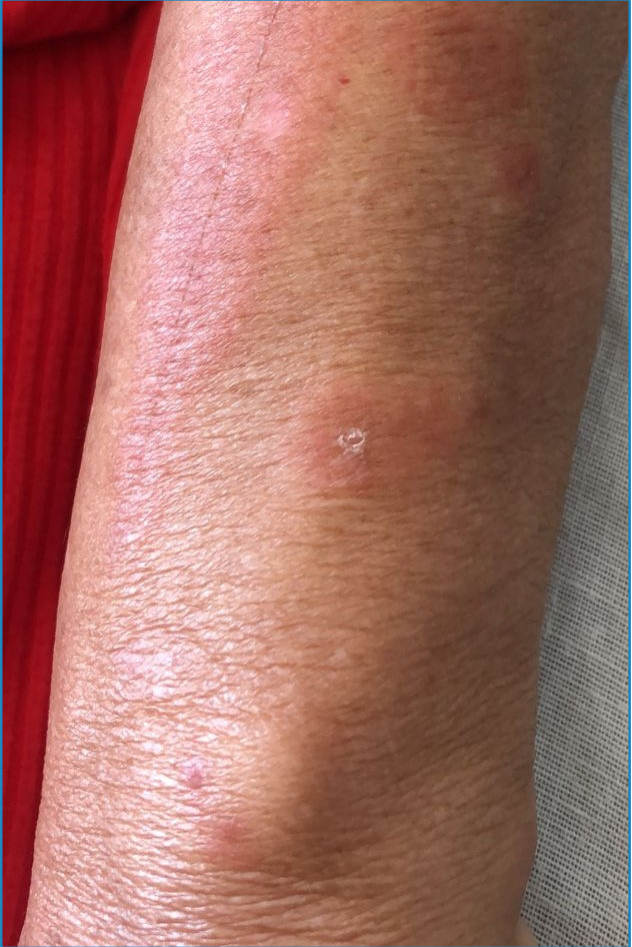
Photograph showing a macular “target” lesion.

An 81-year-old woman was admitted for evaluation of cough, fever, and mild dyspnea, which required supplemental oxygen inhaler use. COVID-19 polymerase chain reaction assay showed positive results 3 days after the onset of symptoms. Nirmatrelvir/ritonavir therapy was initiated 4 days after onset of symptoms and was continued for 5 days. An acute cutaneous eruption of reddish annular macules suggestive of EM was observed on the patient’s arms the day following completion of the medication regimen (Figures 1 and 2). Histopathological examination of the lesions was not performed. Prednisone (1 mg/kg) therapy was initiated, which led to rapid improvement. 

## DISCUSSION

To our knowledge, this is the first report of EM that showed a positive outcome in a patient with COVID-19, who was treated with nirmatrelvir/ritonavir. 

Previous studies have reported EM after COVID-19 and lopinavir/ritonavir administration^9^. Likewise, EM associated with COVID-19 has been described in the medical literature, both in patients aged <30 years and in those aged >55 years^7^.

Our patient did not undergo histopathological examination; however, the temporal relationship between the appearance of the lesion after medication initiation and improvement after discontinuation of therapy reinforces the contribution of nirmatrelvir/ritonavir to EM in this case. The Naranjo Adverse Drug Reaction Probability Scale^10^ score in our patient was 5, which indicates a probable adverse reaction and strengthens our suspicion.

We could not differentiate whether EM was due to a viral infection or secondary to the use of the aforementioned medications in our patient, which serves as a limitation of this study.

## CONCLUSIONS

In this case report, we describe a rare skin reaction following the use of nirmatrelvir/ritonavir. Our findings will serve as guidelines for early detection of this complication and can aid with the establishment of the most suitable treatment in such cases.
